# Association of adverse birth outcomes with in vitro fertilization after controlling infertility factors based on a singleton live birth cohort

**DOI:** 10.1038/s41598-022-08707-x

**Published:** 2022-03-16

**Authors:** Huiting Yu, Zhou Liang, Renzhi Cai, Shan Jin, Tian Xia, Chunfang Wang, Yanping Kuang

**Affiliations:** 1 Vital Statistical Department, Institute of Health Information, Shanghai Municipal Center for Disease Control and Prevention, Shanghai, 200336 People’s Republic of China; 2 School of Public Health, Fudan University, Shanghai, People’s Republic of China; 3 Department of Assisted Reproduction, Shanghai Ninth People’s Hospital, Shanghai Jiao Tong University School of Medicine, Zhizaoju Road No. 639, Shanghai, 200011 People’s Republic of China

**Keywords:** Epidemiology, Reproductive disorders, Intrauterine growth, Preterm birth

## Abstract

Infants conceived with in vitro fertilization (IVF) are exposed to underlying infertility and the IVF process. High risks of adverse birth outcomes (ABOs) were observed among these infants, including preterm birth, low birth weight, macrosomia, being large/small for gestational age (LGA/SGA). It is unclear whether the specific etiology of the rise of ABOs among IVF infants is IVF technology itself or underlying infertility. A total of 9,480 singletons conceived with IVF and 1,952,419 singletons from the general population were obtained in this study. Multivariable logistic regression model was used to assess variations in risk of ABOs according to causes of infertility. Poisson distributions were applied to calculate standardized risk ratios of IVF infants vs. general population after controlling the causes of infertility. Higher risk of preterm birth and low birth weight were observed among parents with polycystic ovary syndrome, endometriosis, uterine and semen abnormalities. Compared to the general population, after excluding the influence of infertility causes, singletons conceived with IVF were at higher risk of macrosomia (SRR = 1.28, 95% CI 1.14–1.44) and LGA (SRR = 1.25, 95% CI 1.15–1.35). The higher risk of ABOs in IVF was driven by both IVF treatments and infertility, which is important for improving IVF treatments and the managing pregnancies and child development.

## Introduction

As lifestyles and living environments continue to evolve, infertility is increasing globally, currently affecting more than one-sixth of couples worldwide^1–4^. Assisted reproductive technology (ART), especially in vitro fertilization (IVF), is widely used for infertility treatment worldwide. Infants conceived by ART have been reported to account for 4–10% of live births in developed countries^5,6^.

Studies have shown that IVF is associated with an increased risk of adverse birth outcomes (ABOs), such as 1.2–2 times the risk of preterm birth (PTB) and low birth weight (LBW)^7^, 1.2 times of small for gestational age (SGA)^8^, and 1.5 times the risk of congenital abnormality^9^. Infants conceived with IVF are affected by underlying infertility factors in addition to the IVF process. However, it remains unclear whether the association of IVF and ABOs originates from the IVF process or the infertility factors. A systematic review and meta-analysis reported that women with endometriosis had a higher risk of PTB and a similar SGA risk after IVF compared to women without endometriosis^10^. A cohort study of singletons found a higher risk of PTB and large for gestational age (LGA) after IVF in women with polycystic ovary syndrome (PCOS)^11^. However, other studies reported that women with unexplained infertility were not at a higher risk of ABOs following IVF^12^. Infants of subfertile women who conceived naturally had a higher risk of ABOs than infants of fertile women^13,14^. Taking these studies into consideration, it remains unknown whether IVF increases the risk of ABOs when controlling for the influence of the underlying causes of infertility.

The purpose of this study was to further investigate whether the specific cause of infertility affects the birth outcomes of IVF. In addition, we conducted a large population-based cohort study to assess whether IVF increases the risk of ABOs compared to the general population or whether the increased risks are associated with infertility.

## Materials and methods

This population-based retrospective cohort study was approved by the institutional review board of the Shanghai Municipal Centre for Disease Control and Prevention (SCDC), and all methods were performed in accordance with the relevant guidelines and regulations. Following the principles of ethical review in China, it is not necessary to reacquire informed consent from participants for retrospective studies based on population registry information. There, the requirement to obtain written informed consent was waived.

### Assisted reproductive technology data

**Figure 1:**
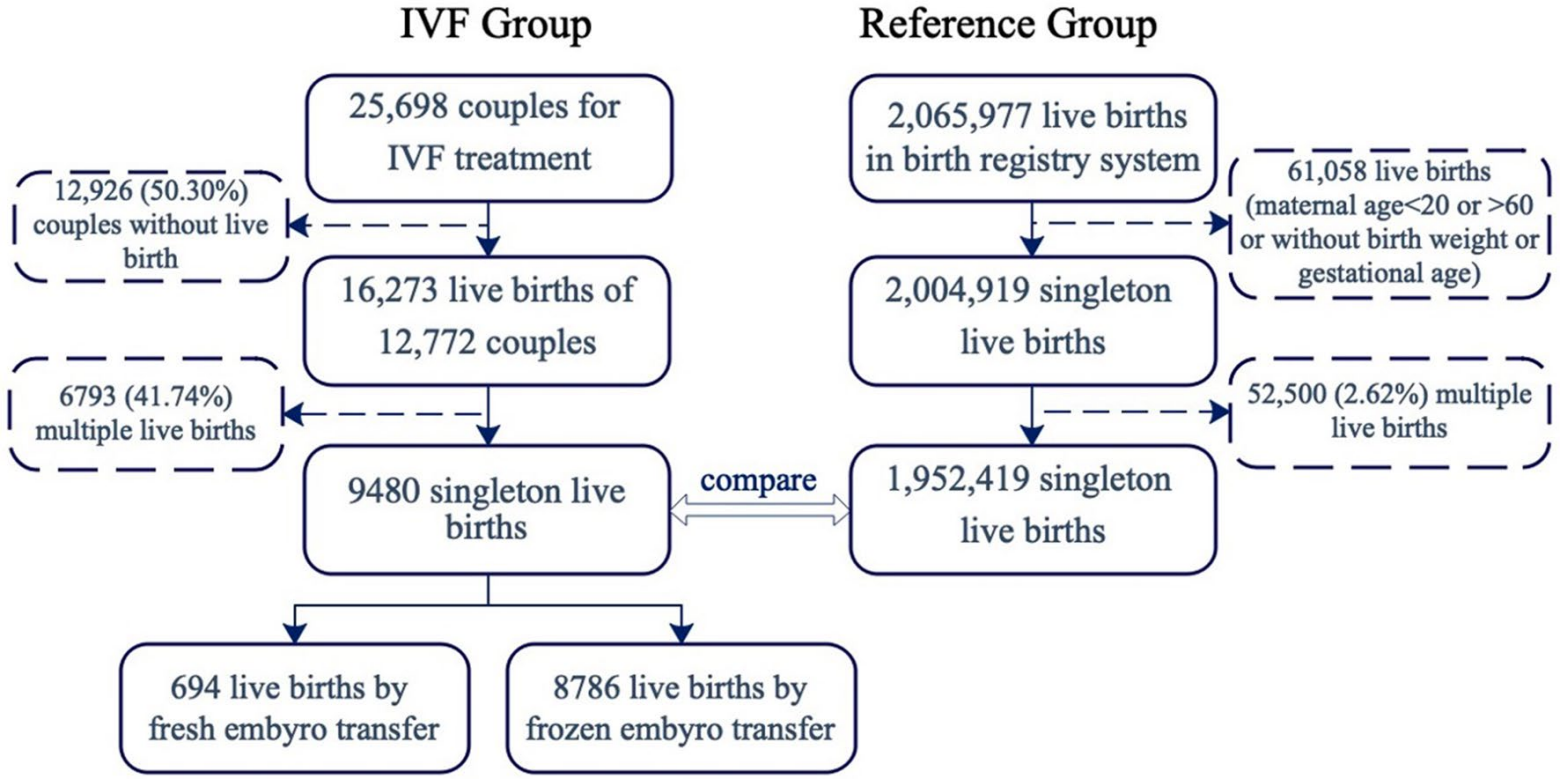
Flow chart of participants included in the study.

Treatment information and birth outcomes of infants conceived with ART were obtained from the Assisted Reproduction Centre of the Ninth People's Hospital affiliated with Shanghai Jiao Tong University of Medicine, Shanghai, China. The centre provided data for infertility treatments performed from January 2008 through December 2017. Out of 25,698 patients (20–60 years old) who visited the centre for IVF treatment, 12,772 (49.70%) delivered one or more liveborn infants (Fig. 1). After excluding 6793 (41.74%) multiple-birth deliveries, the final sample included 9,480 singleton infants conceived with IVF (694 fresh and 8786 frozen).

As per the technical standard for Human assisted reproduction under the Ministry of Health of China^15–17^, details of baseline infertility investigations, ART treatments, and resultant births through ART were recorded for each participant. In addition, data on the treatment period, maternal age, pregestational body mass index (BMI), infertility duration, cause of infertility, fresh or frozen embryo transfer, and live birth occurrence were obtained for this study.

### Birth registry data

Birth information from the whole population came from the birth registry system of SCDC, which was established in 2003 and provides all hospitals with authorized delivery services in Shanghai. To ensure comparability with ART data, 2,065,977 live births were collected from the birth registry system during 2008–2017 (Fig. 1). Due to the mother’s age (< 20 or > 60) or insufficient data (no birth weight or gestational age), 61,058 (2.96%) live births were excluded. After excluding 52,500(2.62%) multiple-birth deliveries, 1,952,419 singleton births were set up as the reference group for analysis. The birth information includes the date of birth, infant sex, birth weight, gestational age at birth, embryo number, mode of delivery, gravidity, parity, and socio-demographic characteristics of the parents.

### Definitions

To compare the gestational age with that of the general population, the gestational age for an IVF infant was calculated as the interval from the date of embryo transfer to the date of birth plus 14 days and the duration of in vitro embryo culture^18^. The main ABOs concerned in this study included PTB, LBW, macrosomia, LGA, and SGA. According to the World Health Organization, PTB is defined as delivery before 37 completed weeks of gestation (or 259 days). LBW is defined as a birth weight of less than 2500 g and macrosomia is defined as a birth weight of over 4000 g. According to the China national population-based sex-specific reference curve for normal fetal growth, SGA and LGA are defined as having a birth weight < 10th or > 90th percentile for gestational age, respectively^19^. In the general population, 20–29 years old is the best childbearing age, so the age group of 20–29 years old was set as the reference group^20^. The remaining ages were divided into groups of 5 years.

### Statistical analysis

Maternal and neonatal characteristics in the IVF group and reference group were described and the chi-square test was used to compare the difference between the IVF group and the general population. We applied a multivariable logistic regression model to evaluate the influence of parental and treatment-related factors on the risk of PTB, LBW, macrosomia, SGA, and LGA among infants conceived with IVF. The factors evaluated in the model included the period of birth, infant sex, maternal age, pregestational BMI, parity, cause of infertility, years of infertility, number of previous procedures involving ART, fresh or frozen embryo transfer cycles, and whether the sperm was from the husband or a donor. Causes of infertility were identified as semen abnormalities (including oligospermia, azoospermia, and teratozoospermia), endometriosis, PCOS, other ovulation failures, tubal factors, and uterine factors (including malformation or pathological uterine or abnormal cervix). Cases where no cause could be found were defined as unexplained infertility. Each diagnosis was set as a binary variable (Yes/No) in the multivariable logistic regression model.

The number of PTB, LBW, macrosomia, SGA, and LGA infants conceived with IVF were compared with expected numbers following a Poisson distribution, which were calculated based on the Shanghai birth registry data and adjusted according to the birth year, maternal age, infant sex, and parity distributions of women conceived with IVF. Standardized risk ratios (SRR) for PTB, LBW, macrosomia, SGA, and LGA were calculated by dividing the observed numbers by the expected numbers and estimated the 95% confidence intervals (CIs).

Additional analyses were performed on the subsamples to distinguish the effect of IVF procedures from subfertility characteristics and the cause of infertility. First, according to the multivariable logistic regression model mentioned above, the subsample was restricted to infants from couples with normal BMI and without infertility factors, including semen abnormalities, endometriosis, polycystic ovary syndrome and uterine factor, which could have affected the birth outcomes. Second, the subsample was limited to infants born to couples with normal BMI and without any apparent cause of infertility (i.e., the unexplained cause group). Third, the subsample was restricted to infants born to couples with a sole diagnosis of tubal disease and with normal BMI; since these infants were considered to be more likely to be conceived with healthy gametes. The statistical and data analysis software package SAS 9.4 was used for data analysis. A value of p < 0.05 was considered to indicate statistical significance.

## Results

### Maternal and neonatal characteristics

**Figure 2:**
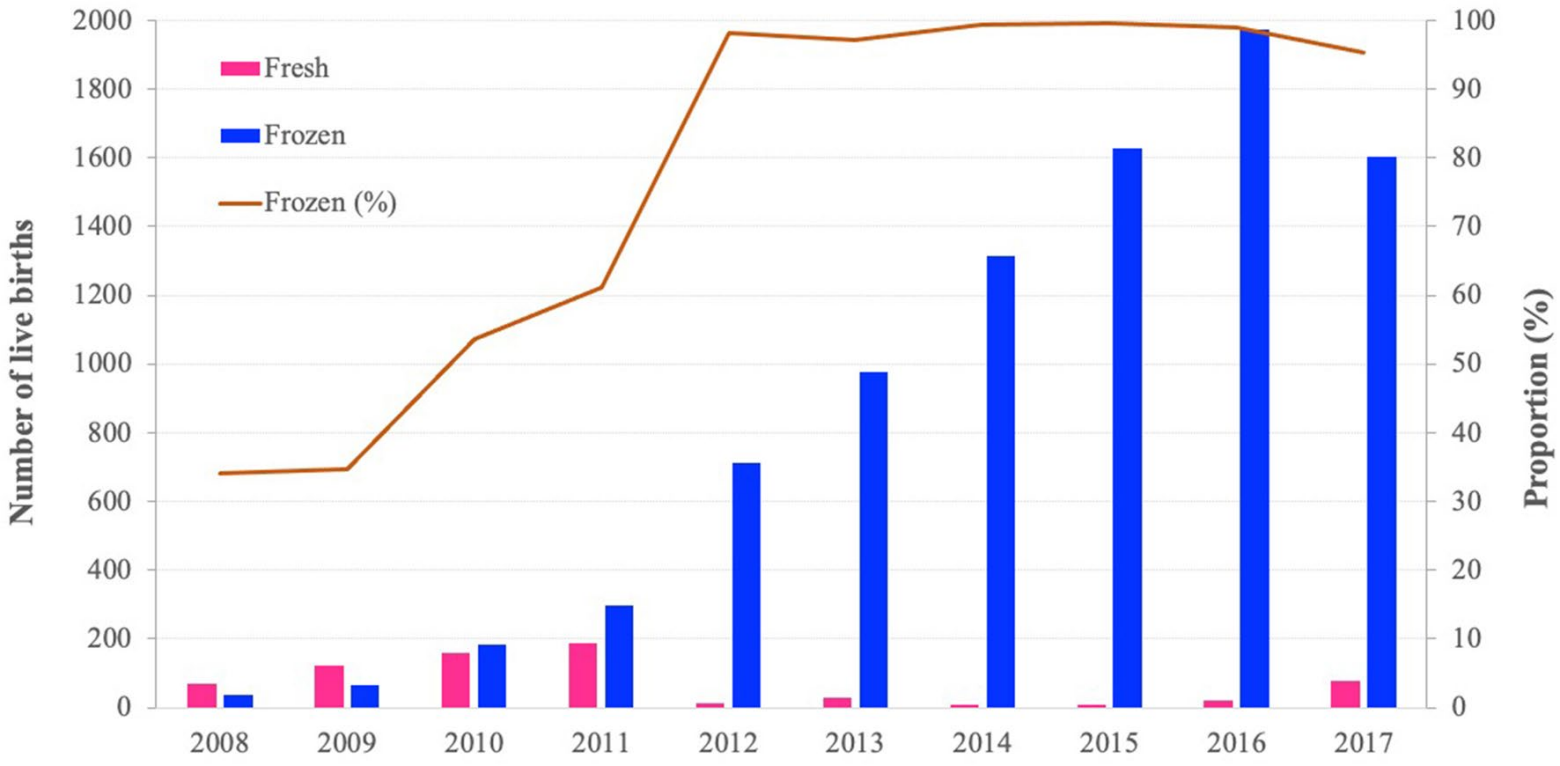
The trends of live births by fresh- and frozen-embryo transfer. From 2008 to 2017, there was increased in trend in the proportion of frozen-embryo transfer (Cochran−Armitage trend test χ^2^ = 41.06, P < 0.0001).

The number of infants conceived with IVF had increased dramatically in recent years, and the utilization rate of frozen embryo transfer had increased from 34% in 2008 to 98% in 2012 and remained above 95% until 2017. (Fig. 2). Maternal and neonatal characteristics showed great differences between the general population and those treated with IVF. The maternal age of women who had conceived with IVF (Mean = 32.80, 95% CI 32.72–32.88) tended to be higher than that in the general population (Mean = 28.15, 95% CI 28.11–28.19) (Table 1). Moreover, women who conceived with IVF were more likely to undergo a caesarean section (76.75% vs. 47.60%, P < 0.0001). The sex ratios at birth of IVF (114.09) and the general population (112.54) were much higher than the United Nations recommendation of 103–107. The average gestational age and birth weight of infants conceived with IVF did not differ significantly from the general population. However, the incidence of PTB (6.59%) and LBW (4.14%) was much higher than that in the general population (4.57 and 2.84%, P < 0.0001). The incidence of macrosomia (8.66%) and LGA (17.82%) was also higher than that in the general population (6.97% and 14.44%, P < 0.0001), while there was no statistically significant difference in the incidence of SGA between IVF (4.98%) and the general population (5.09%, P = 0.6388).

**Table 1:** Maternal and neonatal characteristics of IVF infants and general population.

Variable	Variable level	IVF total		Fresh IVF		Frozen IVF		General population		General population VS. IVF
		N	%	N	%	N	%	N	%	P value
Maternal age	Mean/Std	32.80	4.07	32.62	3.88	32.82	4.09	28.15	4.49	
	20–29 years	2507	26.45	187	26.95	2320	26.41	1,269,832	65.04	< 0.0001
	30–34 years	4232	44.64	300	43.23	3932	44.75	502,893	25.76	
	35–39 years	2268	23.92	189	27.23	2079	23.66	155,388	7.96	
	≥ 40 years	473	4.99	18	2.59	455	5.18	24,306	1.24	
	Missing	0	–	0	–	0	–	0	–	
Maternal education level	Postgraduate	386	5.8	7	4.64	379	5.83	115,866	5.93	< 0.0001
	Graduate	3963	59.59	99	65.56	3864	59.45	899,125	46.05	
	Middle School	2201	33.09	43	28.48	2158	33.2	383,472	19.64	
	Primary School	101	1.52	2	1.32	99	1.52	553,943	28.37	
	Missing	2829	–	543	–	2286	–	13	–	
Parity	0	8624	90.97	630	90.78	7994	90.99	1,315,266	67.37	< 0.0001
	1	808	8.52	61	8.79	747	8.5	577,600	29.58	
	≥ 2	48	0.51	3	0.43	45	0.51	59,553	3.05	
	Missing	0	–	0	–	0	–	0	–	
Delivery mode	Vaginal	2202	23.25	136	19.65	2066	23.54	1,022,996	52.40	< 0.0001
	Cesarean	7268	76.75	556	80.35	6712	76.46	929,423	47.60	
	Missing	10	–	2	–	8	–	0	–	
Sex	Boy	5052	53.29	355	51.15	4697	53.46	1,033,763	52.95	0.5041
	Girl	4428	46.71	339	48.85	4089	46.54	918,656	47.05	
	Sex ratio	–	114.09	–	104.71	–	114.87	–	112.54	
	Missing	0	–	0	–	0	–	0	–	
Gestational age	Mean/Std	38.34	1.65	38.30	1.67	38.35	1.65	38.89	1.49	
	Missing	0	–	0	–	0	–	0	–	
PTB	625	6.59	37	5.33	588	6.69	89,242	4.57	< 0.0001	
Birth weight	Mean/Std	3347.93	502.67	3320.15	495.44	3350.12	503.18	3347.09	453.15	
	Missing	0	–	0	–	0	–	0	–	
LBW		392	4.14	25	3.6	367	4.18	55,500	2.84	< 0.0001
Macrosomia		821	8.66	56	8.07	765	8.71	136,032	6.97	< 0.0001
SGA		472	4.98	28	4.03	444	5.05	99,282	5.09	0.6388
LGA		1689	17.82	119	17.15	1570	17.87	282,006	14.44	< 0.0001

### Risk factors of ABOs in IVF

We found a decreased trend of PTB (OR 0.94, 95% CI 0.90–0.98) and LGA (OR 0.97, 95% CI 0.94–1.00) in IVF in recent years (Table 2). In this study, maternal age was not a significant risk factor for ABOs; however, overweight (24 ≤ BMI < 28) and obesity (BMI ≥ 28) was associated with a higher risk of PTB (OR 1.42, 95% CI 1.15–1.77; OR 1.68, 95% CI 1.16–2.44, respectively).

**Table 2:** Risk factors of adverse birth outcomes in singletons conceived with IVF.

Variable	Variable level	PTB			LBW			Macrosomia			SGA			LGA		
		N	Rate (%)	OR (95%CI)	N	Rate (%)	OR (95%CI)	N	Rate (%)	OR (95%CI)	N	Rate (%)	OR (95%CI)	N	Rate (%)	OR (95%CI)
Year of birth	Change per year	–	–	**0.94(0.90,0.98)**	–	–	0.98(0.93,1.04)	–	–	0.99(0.95,1.03)	–	–	1.01(0.96,1.06)	–	–	**0.97(0.94,1.00)**
Maternal age	20–29 years	166	6.76	Ref	101	4.11	Ref	226	9.20	Ref	127	5.17	Ref	451	18.36	Ref
	30–34 years	263	6.35	0.87(0.71,1.07)	172	4.15	0.95(0.74,1.23)	361	8.72	0.88(0.73,1.05)	192	4.64	0.93(0.73,1.17)	730	17.63	0.90(0.78,1.02)
	35–39 years	142	6.42	0.86(0.67,1.11)	87	3.93	0.89(0.65,1.22)	**187**	**8.46**	**0.80(0.64,0.99)**	116	5.25	1.11(0.84,1.47)	395	17.87	0.86(0.73,1.01)
	≥ 40 years	45	9.78	1.29(0.88,1.88)	21	4.57	1.03(0.61,1.72)	**34**	**7.39**	**0.62(0.41,0.92)**	24	5.22	1.17(0.72,1.87)	81	17.61	0.77(0.58,1.02)
Pregestational BMI	< 18.5	65	6.01	1.02(0.77,1.34)	39	3.60	0.97(0.69,1.37)	**45**	**4.16**	**0.48(0.35,0.65)**	**86**	**7.95**	**1.84(1.43,2.37)**	**103**	**9.52**	**0.50(0.40,0.62)**
	18.5–23.9	395	6.08	Ref	247	3.80	Ref	518	7.98	Ref	292	4.50	Ref	1114	17.16	Ref
	24–28	**120**	**8.82**	**1.42(1.15,1.77)**	**72**	**5.29**	**1.41(1.07,1.85)**	**200**	**14.70**	**2.03(1.70,2.43)**	68	5.00	1.15(0.88,1.51)	**360**	**26.45**	**1.75(1.52,2.01)**
	≥ 28	**36**	**10.84**	**1.68(1.16,2.44)**	**23**	**6.93**	**1.84(1.17,2.90)**	**45**	**13.55**	**1.89(1.35,2.64)**	13	3.92	0.92(0.52,1.63)	**80**	**24.10**	**1.55(1.19,2.03)**
Gender	Boy	347	7.03	Ref	184	3.73	Ref	516	10.45	Ref	228	4.62	Ref	882	17.86	Ref
	Girl	269	6.22	0.89(0.75,1.05)	**197**	**4.55**	**1.23(1.00,1.52)**	**292**	**6.75**	**0.62(0.53,0.72)**	231	5.34	1.17(0.97,1.42)	775	17.91	1.01(0.91,1.12)
Parity	0	542	6.43	Ref	350	4.15	Ref	721	8.55	Ref	427	5.06	Ref	1479	17.54	Ref
	1	**70**	**8.89**	**1.41(1.07,1.85)**	29	3.68	0.94(0.63,1.40)	82	10.42	1.18(0.92,1.52)	31	3.94	0.77(0.52,1.13)	166	21.09	1.22(1.01,1.47)
	≥ 2	4	8.51	1.31(0.46,3.70)	2	4.26	1.09(0.26,4.59)	5	10.64	1.19(0.46,3.06)	1	2.13	0.42(0.06,3.06)	12	25.53	1.58(0.81,3.08)
Year of infertility	< 5 years	424	6.32	Ref	265	3.95	Ref	583	8.69	Ref	339	5.05	Ref	1187	17.69	Ref
	5–10 years	162	7.59	1.15(0.95,1.40)	98	4.59	1.11(0.87,1.42)	189	8.86	0.99(0.82,1.18)	101	4.74	0.96(0.76,1.22)	389	18.24	0.99(0.87,1.13)
	≥ 10 years	30	7.08	1.01(0.68,1.52)	18	4.25	1.03(0.62,1.72)	36	8.49	0.97(0.67,1.40)	19	4.48	0.88(0.54,1.45)	81	19.10	1.03(0.79,1.34)
Number of failure cycle	0	368	6.37	Ref	228	3.95	Ref	485	8.40	Ref	299	5.18	Ref	1005	17.40	Ref
	1	99	6.10	0.87(0.69,1.11)	57	3.51	0.86(0.63,1.17)	153	9.43	1.17(0.96,1.43)	69	4.25	0.80(0.60,1.05)	300	18.48	1.07(0.92,1.24)
	2	69	8.03	1.20(0.91,1.58)	43	5.01	1.24(0.88,1.75)	78	9.08	1.16(0.90,1.51)	40	4.66	0.88(0.62,1.24)	159	18.51	1.10(0.91,1.34)
	≥ 3	80	7.94	1.16(0.88,1.51)	53	5.26	1.29(0.93,1.79)	92	9.13	1.17(0.91,1.50)	51	5.06	0.97(0.70,1.33)	193	19.15	1.16(0.96,1.38)
Sperm	Husband sperm	608	6.66	Ref	372	4.08	Ref	799	8.76	Ref	449	4.92	Ref	1635	17.92	Ref
	Donor sperm	8	5.63	0.84(0.40,1.78)	9	6.34	1.42(0.68,2.93)	9	6.34	0.73(0.36,1.49)	10	7.04	1.45(0.72,2.90)	22	15.49	0.80(0.49,1.29)
Embryo transfer	Fresh	37	5.51	Ref	25	3.72	Ref	56	8.33	Ref	28	4.17	Ref	118	17.56	Ref
	Frozen	**579**	**6.74**	**1.51(1.03,2.21)**	356	4.14	1.14(0.72,1.81)	752	8.75	1.07(0.77,1.47)	431	5.01	1.20(0.78,1.84)	1539	17.91	1.13(0.90,1.43)
Unexplained	No	603	6.63	Ref	373	4.10	Ref	795	8.74	Ref	447	4.91	Ref	1626	17.87	Ref
	Yes	13	7.83	1.29(0.69,2.41)	8	4.82	1.51(0.69,3.29)	13	7.83	0.74(0.40,1.36)	12	7.23	1.45(0.75,2.81)	31	18.67	0.92(0.60,1.42)
Semen abnormalities	No	515	6.59	Ref	309	3.95	Ref	688	8.81	Ref	385	4.93	Ref	1397	17.88	Ref
	Yes	101	6.95	1.08(0.86,1.35)	**72**	**4.95**	**1.31(1.00,1.71)**	120	8.25	0.90(0.74,1.11)	74	5.09	1.04(0.80,1.35)	260	17.88	0.99(0.85,1.15)
Endometriosis	No	532	6.52	Ref	325	3.98	Ref	726	8.89	Ref	400	4.90	Ref	1484	18.18	Ref
	Yes	**84**	**7.62**	**1.29(1.01,1.65)**	**56**	**5.08**	**1.37(1.01,1.85)**	82	7.43	0.85(0.66,1.08)	59	5.35	1.05(0.78,1.40)	173	15.68	0.88(0.73,1.05)
PCOS	No	542	6.35	Ref	342	4.01	Ref	746	8.74	Ref	432	5.06	Ref	1517	17.77	Ref
	Yes	**74**	**10.11**	**1.65(1.25,2.18)**	39	5.33	1.30(0.90,1.87)	**62**	**8.47**	**0.74(0.55,0.99)**	27	3.69	0.74(0.49,1.12)	140	19.13	0.90(0.73,1.10)
Other ovulation failure	No	588	6.60	Ref	363	4.08	Ref	779	8.75	Ref	436	4.90	Ref	1605	18.02	Ref
	Yes	28	7.76	1.28(0.85,1.92)	18	4.99	1.36(0.83,2.24)	29	8.03	0.88(0.59,1.32)	23	6.37	1.33(0.85,2.08)	52	14.40	0.75(0.55,1.02)
Tubal factor	No	112	7.87	Ref	67	4.71	Ref	128	9.00	Ref	79	5.55	Ref	265	18.62	Ref
	Yes	504	6.43	0.95(0.74,1.21)	314	4.00	1.03(0.76,1.40)	680	8.67	0.88(0.70,1.11)	380	4.84	0.92(0.69,1.23)	1392	17.75	0.89(0.76,1.06)
Uterine factor	No	503	6.30	Ref	302	3.78	Ref	702	8.80	Ref	391	4.90	Ref	1438	18.02	Ref
	Yes	**113**	**8.78**	**1.42(1.14,1.77)**	**79**	**6.14**	**1.60(1.23,2.08)**	106	8.24	0.89(0.72,1.11)	68	5.28	1.08(0.83,1.42)	219	17.02	0.90(0.77,1.06)
Others*	No	599	6.64	Ref	372	4.13	Ref	783	8.69	Ref	449	4.98	Ref	1609	17.85	Ref
	Yes	17	6.75	1.05(0.63,1.75)	9	3.57	0.80(0.40,1.59)	25	9.92	1.21(0.78,1.87)	10	3.97	0.68(0.35,1.30)	48	19.05	1.18(0.85,1.65)

*Others: including chromosomal abnormality, immune factors, pituitary lesions and sexual dysfunction.

Statistical differences are shown in bold.

LBW (OR 1.41, 95% CI 1.07–1.85; OR 1.84, 95% CI 1.17–2.90, respectively), macrosomia (OR 2.03, 95% CI 1.70–2.43; OR 1.89, 95% CI 1.35–2.64, respectively), and LGA (OR 1.75, 95% CI 1.52–2.01; OR 1.55, 95% CI 1.19–2.03, respectively), while lower pregestational BMI (< 18.5) was associated with SGA (OR 1.84, 95% CI 1.43–2.37). In addition, female infants had a higher risk of LBW (OR 1.23, 95% CI 1.00–1.52) and a lower risk of macrosomia (OR 0.62, 95% CI0.53–0.72). Frozen embryo transfer was associated with a higher risk of PTB (OR 1.51, 95% CI 1.03–2.21), but there was no statistically significant difference in LBW, macrosomia, SGA and LGA.

Regarding the causes of infertility, semen abnormalities (OR 1.31, 95% CI 1.00–1.71) were related to a higher risk of LBW; endometriosis was related to a higher risk of PTB (OR 1.29, 95% CI 1.01–1.65) and LBW (OR 1.37, 95% CI 1.01–1.85); PCOS was linked to a higher risk of PTB (OR 1.65, 95% CI 1.25–2.18) and lower risk of macrosomia (OR 0.74, 95% CI 0.55–0.99); and uterine factor infertility was related to a higher risk of PTB (OR 1.42, 95% CI 1.14–1.77) and LBW (OR 1.60, 95% CI 1.23–2.08). There was no statistically significant difference in the incidence of ABOs between women with or without tubal infertility.

### Comparison of ABOs risk between IVF and the general population

Compared with the general population, singletons conceived with IVF had a higher risk of PTB (SRR = 1.15, 95% CI 1.06–1.24), LBW (SRR = 1.12, 95% CI 1.02–1.24), macrosomia (SRR = 1.34, 95% CI 1.26–1.44), and LGA (SRR = 1.26, 95% CI 1.20–1.32) (Table 3). We stratified IVF procedures based on fresh and frozen embryo transfer cycles and found that the risks of all ABOs categories were still significantly higher in frozen cycles, whereas only the risks of LGA (SRR = 1.21, 95% CI 1.01–1.45) remained higher in fresh cycles.

**Table 3:** Observed and expected cases of adverse birth outcomes among infants conceived with IVF in mothers with normal BMI.

ABOs	Group	IVF				IVF-Fresh				IVF-Frozen			
		Total no	No. of events		SRR* (95%CI)	Total no	No. of events		SRR* (95%CI)	Total no	No. of events		SRR* (95%CI)
			Observed	Expected^#^			Observed	Expected^#^			Observed	Expected^#^	
PTB	IVF Total	**9480**	**625**	**543.2**	**1.15(1.06,1.24)**	694	37	39.8	0.93(0.67,1.28)	**8786**	**588**	**503.4**	**1.17(1.08,1.27)**
	Excluding infertility factors affecting ABOs ^$^	3787	229	217.0	1.06(0.93,1.20)	336	16	19.3	0.83(0.51,1.36)	3451	213	197.7	1.08(0.94,1.23)
	Unexplained infertility cause	111	8	6.4	1.26(0.63,2.52)	–	–	–	–	99	8	5.7	1.41(0.71,2.82)
	Tubal factor infertility	3333	199	191.0	1.04(0.91,1.20)	286	13	16.4	0.79(0.46,1.37)	3047	186	174.6	1.07(0.92,1.23)
LBW	IVF Total	**9480**	**392**	**348.9**	**1.12(1.02,1.24)**	694	25	25.5	0.98(0.66,1.45)	**8786**	**367**	**323.3**	**1.14(1.02,1.26)**
	Excluding infertility factors affecting ABOs ^$^	3787	133	139.4	0.95(0.81,1.13)	336	12	12.4	0.97(0.55,1.71)	3451	121	127.0	0.95(0.80,1.14)
	Unexplained infertility cause	111	3	4.1	0.73(0.24,2.28)	–	–	–	–	99	3	3.6	0.82(0.27,2.55)
	Tubal factor infertility	3333	115	122.7	0.94(0.78,1.13)	286	10	10.5	0.95(0.51,1.77)	3047	105	112.1	0.94(0.77,1.13)
Macrosomia	IVF total	**9480**	**821**	**610.5**	**1.34(1.26,1.44)**	694	56	44.7	1.25(0.96,1.63)	**8786**	**765**	**565.8**	**1.35(1.26,1.45)**
	Excluding infertility factors affecting ABOs ^$^	**3787**	**308**	**243.9**	**1.26(1.13,1.41)**	336	23	21.6	1.06(0.71,1.60)	**3451**	**285**	**222.2**	**1.28(1.14,1.44)**
	Unexplained Infertility cause	111	10	7.1	1.40(0.75,2.60)	12	1	0.8	1.29(0.18,9.19)	99	9	6.4	1.41(0.73,2.71)
	Tubal factor infertility	**3333**	**271**	**214.6**	**1.26(1.12,1.42)**	286	22	18.4	1.19(0.79,1.81)	**3047**	**249**	**196.2**	**1.27(1.12,1.44)**
SGA	IVF total	9480	472	486.3	0.97(0.89,1.06)	694	28	35.6	0.79(0.54,1.14)	8786	444	450.7	0.99(0.90,1.08)
	Excluding infertility factors affecting ABOs ^$^	3787	174	194.3	0.90(0.77,1.04)	336	16	17.2	0.93(0.57,1.52)	3451	158	177.0	0.89(0.76,1.04)
	Unexplained infertility cause	111	7	5.7	1.23(0.59,2.58)	12	1	0.6	1.62(0.23,11.53)	99	6	5.1	1.18(0.53,2.63)
	Tubal factor infertility	3333	149	171.0	0.87(0.74,1.02)	286	12	14.7	0.82(0.46,1.44)	3047	137	156.3	0.88(0.74,1.04)
LGA	IVF total	**9480**	**1689**	**1342.4**	**1.26(1.20,1.32)**	**694**	**119**	**98.3**	**1.21(1.01,1.45)**	**8786**	**1570**	**1244.1**	**1.26(1.20,1.33)**
	Excluding infertility factors affecting ABOs ^$^	**3787**	**662**	**536.2**	**1.23(1.14,1.33)**	336	53	47.6	1.11(0.85,1.46)	**3451**	**609**	**488.7**	**1.25(1.15,1.35)**
	Unexplained infertility cause	111	22	15.7	1.40(0.92,2.13)	12	1	1.7	0.59(0.08,4.18)	99	21	14.0	1.50(0.98,2.30)
	Tubal factor infertility	**3333**	**589**	**472.0**	**1.25(1.15,1.35)**	286	47	40.5	1.16(0.87,1.54)	**3047**	**542**	**431.5**	**1.26(1.15,1.37)**

^#^The number of expected cases was calculated by applying the rates of PTB, LBW, macrosomia, SGA and LGA from the birth registry data to the population of infants conceived with assisted reproductive technology. The values were adjusted to account for differences in the distributions of year of birth, maternal age, infant sex and parity between the two populations.

*SRR standardized risk ratio.

^$^Infertility factors affecting ABOs meant infertility factors that influenced the birth outcomes, including semen abnormalities, endometriosis, polycystic ovary syndrome and uterine factor infertility.

Statistical differences are shown in bold.

In the subgroup analyses, we found that the risks of PTB and LBW no longer increased. However, the risks of macrosomia and LGA remained elevated in analyses restricted to subgroups of the infants conceived by women with a sole diagnosis of tubal factor infertility (macrosomia, SRR = 1.26, 95% CI 1.12–1.42; LGA, SRR = 1.25, 95% CI 1.15–1.35) or by women without infertility factors (semen abnormalities, endometriosis, PCOS, and uterine factor infertility) (macrosomia, SRR = 1.26, 95% CI 1.13–1.41; LGA, SRR = 1.23, 95% CI 1.14–1.33). The risk of LGA (SRR = 1.50, 95% CI 0.98–2.30) was not statistically significant in the group of women diagnosed with unexplained infertility, but the sample size (n = 99) was relatively small.

## Discussion

With the wide application of IVF to treat infertility, a large number of ABOs have been observed among infants conceived with IVF^8,9,18,21^. This large sample study demonstrated that singleton infants conceived with IVF were at a higher risk of ABOs relative to singletons in the general population of Shanghai. These risks could not be explained by the known differences between the two populations in the distribution of sex of infants, maternal age, maternal parity, or maternal BMI. Factors causing infertility, such as semen abnormalities, endometriosis, PCOS, and uterine factors were associated with PTB and abnormal birth weight. However, the increased risks of macrosomia and LGA remained significant when the sample was restricted to (1) parents without infertility factors affecting ABOs wherein the mother has a normal BMI, (2) mothers who have infertility caused by a fallopian tube abnormality. Therefore, this study suggests that the increased risk of birth weight among singletons conceived with IVF may be associated with treatments for infertility. Although the risk of macrosomia and LGA was higher than that in the general population, the incidence of LGA showed a decreasing trend across the entire cohort of IVF cycles, which was also observed in Israel and Sweden^22,23^.

Consistent with previous studies, our study showed that low pregestational BMI (< 18.5) increased the risk of SGA, and being overweight or obese (BMI > 24) before pregnancy increased the risk of PTB, LBW, macrosomia, and LGA^24,25^. BMI abnormalities are more common in the infertile population and may be related to ovulation failure, irregular menses, poor oocyte quality, and hormonal imbalances^26–28^. Additionally, abnormal BMI may be a risk factor for abnormal birth weight in infants conceived with IVF. Therefore, it is necessary to eliminate the influence of abnormal BMI to reveal the real impact of IVF on ABOs.

This study found that PCOS, endometriosis and uterine factors were associated with a higher risk of PTB. Semen abnormalities, endometriosis, and uterine factors were associated with a higher risk of LBW. The association of PCOS with increased risk of PTB but not LBW is similar to previous studies where women with PCOS were found to have a higher risk of gestational diabetes, hypertensive disorders, PTB, and LGA^11,29^. Moreover, the literature showed that after the correction of hypertensive disorders, the increased risk of PTB was eliminated, and even after the correction of gestational diabetes, the increased risk of LGA remained significant^11^. Endometriosis, which affects 10–15% of reproductive age women, is associated with inflammation, fibrosis, and aberrant angiogenesis^30^. Literature shows that endometriosis is associated with a higher risk of PTB and LBW^10,31^, however, this association is influenced by BMI^32^. In this study, we also found that the association between endometriosis and risk of PTB and LBW increased after adjusting for maternal BMI. This finding suggests that the higher risk is associated with mechanisms specific to endometriosis^33^. Additionally, uterine factors such as uterine defects, uterine inflammation, and cervical insufficiency were also associated with a higher risk of PTB and LBW in this study. This finding is supported by literature showing a relationship between uterine factors and many obstetric complications^34,35^. Some studies have reported that tubal factors increase the risk of PTB and LBW for singletons; the etiological reasons were mainly attributed to inflammation and infections^31,36,37^. Conversely, other studies showed that a unilateral tubal block did not increase the risk of ABOs^38,39^. In this study, we did not find any association between tubal factor infertility and PTB or LBW. This difference may be explained by the epidemiological differences in the cause and severity of tubal infertility; hence, further study of the underlying mechanism is warranted.

Another novel finding of this study was that semen abnormality was associated with a higher incidence of LBW but not PTB. This finding contrasts with that of most other studies, which found no significant association between male infertility factors and increased risk of PTB and LBW compared to unexplained infertility^31^. Moreover, semen parameters did not influence embryo quality or live birth outcomes^40,41^. Another study showed that male-factor infertility has been associated with LBW and LBW at full term in singletons conceived with ART^18^; however, it could not be distinguished whether the effect was from the ART or male-factor infertility. This study found that sperm abnormality, oligospermia, and asthenozoospermia were associated with a higher risk of LBW. The increased risk may be due to sperm DNA damage^42^, but the mechanisms underlying the association remain unclear and warrants further research.

Several additional analyses were performed on the subsamples to distinguish the effects of IVF procedures from underlying characteristics of the patients and the cause of infertility. We found a significant association of macrosomia and LGA with IVF, even when abnormal BMI and infertile causes, such as semen abnormalities, endometriosis, polycystic ovary syndrome and uterine factor infertility for abnormal birth weight were eliminated from the study group. Although increased risk was not observed for fresh embryo transfers, this analysis had greatly reduced sample sizes and should therefore be interpreted with caution. Previous studies found that frozen embryo transfer was associated with increased birth weight and a higher risk of macrosomia and LGA, compared with spontaneous conception and fresh embryo transfer^43,44^; however, potential confounding factors, such as maternal BMI or cause of infertility had not been adjusted in those studies. In this study, after adjusting for confounding factors, singletons born after frozen embryo transfer had a higher rate of LGA and macrosomia compared with the general population. This finding may partly be explained by aspects related to ART procedure, such as improved endometrial reception, higher quality embryos surviving the freezing–thawing process^45^, and the effect of cryoprotectants^46^. The embryos were cultured in cryoprotectants and had undergone freezing and thawing procedures at a critical and vulnerable development stage, which could cause epigenetic changes resulting in a larger gestational size^47^. Currently, the mechanism remains unclear and epigenetic effects in infants conceived with ART require further investigation. While LGA and macrosomia may seem less threatening to infant survival, they are associated with an increased risk of cardiovascular diseases in adulthood^48,49^. Thus, more attention should be paid to the association between IVF and increased risk of LGA and macrosomia, and longer-term follow-up of children’s development is also important.

### Strengths and limitations

One of the strengths of this study is its large sample size and detailed information available on the treatments from the infertility service centre. Additionally, the availability of outcomes from many frozen embryo transfers is higher compared with many previously reported studies. Confounding factors that affect embryonal development and underlying infertility have prevented researchers from evaluating the association between IVF and ABOs for a long time. This study adjusted for the confounding effects of underlying infertility to compare the fetal growth between IVF infants and the general population. Our research results indicated that the risk of macrosomia and LGA in IVF was increased, especially when frozen embryo transfer was used.

This study has some limitations. The general population was used as the reference group; however, the general population may include 1.7–4.0% of infants conceived with ART as it is not possible to accurately identify and exclude these births. Therefore, the association between ABOs and IVF was likely underestimated. This retrospective cohort study covers a relatively long period and may be affected by inevitable changes to routines for data collection, approach to diagnosis, and improved procedures for IVF. For example, such changes mean that there may be effects from changes to ovarian stimulation and oocyte collection methods that are hard to distinguish. We also did not collect data on pregnancy complications that may have affected the incidence of ABOs; however, we did include most risk factors for pregnancy complications, such as maternal age, pregestational BMI, endometriosis, and PCOS.

## Conclusions

This cohort study found that causes of infertility, including endometriosis, PCOS, uterine factors, and semen abnormalities increased the risk of ABOs. After adjusting for these factors, compared with the general population, the risk of LBW and PTB did not increase, but the risk of macrosomia and LGA was still increased. Although we could not exclude the potential effect of pregnancy complications, the findings in this study may nonetheless provide insight for patients seeking ART treatment.
