# Author Correction: Association of adverse birth outcomes with in vitro fertilization after controlling infertility factors based on a singleton live birth cohort

**DOI:** 10.1038/s41598-022-12512-x

**Published:** 2022-05-19

**Authors:** Huiting Yu, Zhou Liang, Renzhi Cai, Shan Jin, Tian Xia, Chunfang Wang, Yanping Kuang

**Affiliations:** 1 Vital Statistical Department, Institute of Health Information, Shanghai Municipal Center for Disease Control and Prevention, Shanghai, 200336 People’s Republic of China; 2 School of Public Health, Fudan University, Shanghai, People’s Republic of China; 3 Department of Assisted Reproduction, Shanghai Ninth People’s Hospital, Shanghai Jiao Tong University School of Medicine, Zhizaoju Road No. 639, Shanghai, 200011 People’s Republic of China

Correction to: *Scientific Reports* https://doi.org/10.1038/s41598-022-08707-x, published online 16 March 2022

In the original version of this Article Huiting Yu and Zhou Liang were omitted as equally contributing authors.

The original Article has been corrected.

